# Multinucleation during *C. trachomatis* Infections Is Caused by the Contribution of Two Effector Pathways

**DOI:** 10.1371/journal.pone.0100763

**Published:** 2014-06-23

**Authors:** Heather M. Brown, Andrea E. Knowlton, Emily Snavely, Bidong D. Nguyen, Theresa S. Richards, Scott S. Grieshaber

**Affiliations:** 1 Department of Oral Biology, University of Florida, Gainesville, Florida, United States of America; 2 Department of Molecular Genetics and Microbiology, Center for Microbial Pathogenesis, Duke University Medical Center, Durham, North Carolina, United States of America; University of California, San Francisco, University of California, Berkeley, and the Children's Hospital Oakland Research Institute, United States of America

## Abstract

*Chlamydia trachomatis* is an obligate intracellular bacterial pathogen and the second leading cause of sexually transmitted infections in the US. Infections cause significant morbidity and can lead to serious reproductive sequelae, including an epidemiological link to increased rates of reproductive cancers. One of the overt changes that infected cells exhibit is the development of genomic instability leading to multinucleation. Here we demonstrate that the induction of multinucleation is not conserved equally across chlamydial species; *C. trachomatis* L2 caused high levels of multinucleation, *C. muridarum* intermediate levels, and *C. caviae* had very modest effects on multinucleation. Our data show that at least two effector pathways together cause genomic instability during infection leading to multinucleation. We find that the highly conserved chlamydial protease CPAF is a key effector for one of these pathways. CPAF secretion is required for the loss of centrosome duplication regulation as well as inducing early mitotic exit. The second effector pathway involves the induction of centrosome position errors. This function is not conserved in three chlamydial species tested. Together these two pathways contribute to the induction of high levels of genomic instability and multinucleation seen in *C. trachomatis* infections.

## Introduction

*Chlamydia trachomatis* causes the most common bacterial sexually transmitted disease (STD) in the developed world, with an annual estimated 4 million cases occurring in the United States alone. [1], [2]. Left untreated, these infections can lead to pelvic inflammatory disease, ectopic pregnancy, and infertility [3]. Additionally, there are numerous reports linking chlamydial STD to cervical and ovarian cancers [4]–[7].

*C. trachomatis* infection of vertebrate cells results in a dramatic induction of multinucleation with up to 80% of infected cells become multinucleated [8]–[10]. In our previous studies we found that induction of genomic instability was a major contributing factor to chlamydial induced multinucleation [10]. From this data, we speculated that the combined effects of centrosome amplification, early mitotic exit and centrosome positioning errors led to observed chromosome segregation errors [11], [12]. These phenotypes are important because multinucleation and genomic instability are common in all solid tumors suggesting a causal link between these phenotypes and cancer formation or progression [13]–[15].

Therefore, in an attempt to further determine the molecular events involved in the induction of genomic instability and multinucleation, we investigated the ubiquity of the induction of these phenotypes across divergent chlamydial species. To this end we compared *C. trachomatis* L2 (Ctr L2), *C. muridarum* (MoPn) and *C. caviae* (GPIC) for their ability to induce multinucleation. MoPn is a mouse-specific pathogen that is evolutionarily closely related to *C. trachomatis*. The genomes of these species have been sequenced, and 98.7% of the genes are conserved between *C. trachomatis* and *C. muridarum* [16]–[18]. GPIC is an animal pathogen more distantly related to *C. trachomatis*, with 91.1% of genes conserved between the species [16]–[18]. All three species are well characterized and grow at similar rates, requiring approximately 48 hours to complete the infectious cycle. Additionally, our previous studies implicated the chlamydial effector CPAF as potentially contributing to the induction of multinucleation through its effects on mitotic checkpoint control [10]. Therefore, we also investigated multinucleation induction in a collection of plaque purified chlamydial isolates that contain mutations in the *cpa* gene, rendering them null for CPAF activity or mutations in type II secretion leading to defects in secretion of CPAF.

In this study we demonstrate that the induction of multinucleation is not conserved in all species tested as only cells infected with Ctr L2 and MoPn led to high levels of multinucleation. By dissecting the induction of centrosome amplification, early mitotic exit and centrosome positioning defects in these chlamydial species (GPIC, MoPn, Ctr L2) as well as chlamydial mutants (*cpa* and GspE) we show that all three phenotypes contribute to high levels of multinucleation. The data show that CPAF, which is conserved across all chlamydial species tested, is a key effector required for both early mitotic exit and loss of centrosome duplication regulation, but not centrosome positioning defects. A separate second effector pathway regulates the intimate physical interaction between the chlamydial inclusion and the host microtubule network. This interaction ultimately results in centrosome declustering in Ctr L2 infected cells and to a lesser extent in cells infected with MoPn. However, GPIC infection does not cause significant changes in centrosome clustering. Taken together these data suggest that *Chlamydia trachomatis* possesses two effector pathways that together cause high levels of genomic instability during infection leading to the induction of multinucleation.

## Materials and Methods

### Organisms and Cell Culture

*Chlamydia trachomatis* serovar L2 (LGV 434), *C. muridarum* Nigg strain (referred to as MoPn), *C. caviae* (GPIC) (gift from Ted Hackstadt) were grown in McCoy cells, and EBs were purified by Renografin density gradient centrifugation as previously described [19]. EBs were stored at −80 C until ready for use. *C. trachomatis* L2 CPAF and GspE mutants were generously provided by Rafael Valdivia. CPAF and GspE mutant strains were isolated from a library of chlamydial mutants generated as described [20]. In short, L2-infected Vero cells were exposed to 20 mg/mL ethyl methyl sulfonate (EMS) in PBS for 1 h individual mutants were isolated by plaque purification and arrayed in 96 well plates to generate a library of chlamydial mutants. Genomic DNA was isolated from these mutants and sequenced to determine the genotypes. Homologous recombination between *cpa* mutant M169 and wt L2 was used to generate the rst5 and rst17 isogenic strains as described by Nguyen and Valdivia [20]. The sequences of the mutants are provided in Figure S1. All cell lines were obtained from the American Type Culture Collection. McCoy cells were maintained in DMEM (Gibco), supplemented with 10% FBS (Cellgro) and 10 µg/mL gentamicin (Cellgro). HeLa 229 cells, Neuroblastoma (N1E-115) cells (CRL-2263) and 3T3 cells (CCL-92) were grown in RPMI-1640 (Cellgro) supplemented with 10% fetal bovine serum (FBS) and 10 µg/ml gentamicin.

### Infections

Cells were incubated with *Chlamydia* EBs at an MOI of approximately 3 in Hank's balanced salt solution (HBSS) (Gibco) for 45 minutes at room temperature while rocking. The inoculum was removed and replaced with fresh RPMI-1640 or DMEM containing 10% FBS and 10 µg/ml gentamicin. For the inhibition of centrosome clustering infected cells were treated with the compound griseofulvin. Griseofulvin targets microtubules and prevents the clustering of centrosomes during mitosis [21]–[23]. HeLa cells were infected for 16 hours with chlamydial strains and subsequently treated with 10 µM griseofulvin for an additional 20 hours. Cells were fixed in ice cold methanol and stained.

### Immunohistochemistry

Cells for fluorescent microscopy were grown on 12-mm number 1.5 borosilicate glass coverslips coated with Poly-L-lysine (Sigma). For antibody staining, the coverslips were fixed in ice cold methanol for 10 minutes and incubated with the primary antibody described for each experiment. The antibodies used for these experiments were: mouse monoclonal anti-β-tubulin (Sigma), and mouse monoclonal anti-*γ*-tubulin (Sigma). Nuclear envelope staining was carried out using Lamin A/C mAb (4C11) (Cell Signaling). Ctr L2,- MoPn-, and GPIC-infected cells were stained with platelet poor human serum (reacts with chlamydial LPS) (Sigma). CPAF staining was done using a monoclonal anti-CPAF antibody kindly provide by Dr. Guangming Zhong. To visualize the primary antibodies, AlexaFluor 488 conjugated secondary antibodies against mouse IgG and AlexaFluor 568 conjugated antihuman IgG were used (Molecular Probes/Invitrogen). The far-red fluorescent DNA dye DRAQ5 (Biostatus) or Hoechst was used to visualize nuclei. Images were acquired using a spinning disk confocal system connected to a Leica DMIRB microscope with a 63x oil-immersion objective, equipped with a Photometrics cascade-cooled EMCCD camera, under the control of the Open Source software package µManager (http://www.micro-manager.org/). Images were processed using the image analysis software ImageJ (http://rsb.info.nih.gov/ij/). Images displayed for figures are maximal intensity projections of the 3D data sets, adjusted for brightness and contrast.

### Inclusion measurement

HeLa cells were imaged using confocal microscopy. ImageJ was used to measure the area of the inclusion. We calculated and compared the 2 dimensional areas occupied by the inclusion to simplify quantification. The Z component was comparable and did not significantly change over time. The flat cell shape highly constrained the shape of the inclusion in the Z dimension and significant growth only occurs in the X and Y dimensions.

### Mitotic Index Assays

The mitotic index was calculated by determining the ratio of mitotic cells to the total number of cells present. For the infected populations only infected cells were counted. A minimum of 1500 cells were counted over 20-30 fields and the procedure was repeated 3 times.

### Centrosome Calculations

Centrosome to nucleus distance was calculated by using ImageJ to draw a line from the centrosomes to the closest nucleus. Centrosome spread was calculated by using 3D image stacks to produce a 2D binary image of the centrosomes. The 2D spread was calculated using the ImageJ plug-in “Hull and Circle.” A region of interest was drawn around the centrosomes and the area of the bounding circle was calculated from the minimal fitted polygon.

### Statistical Analyses

Numerical data are presented as the mean ± SEM, and were analyzed by the unpaired t-test or ANOVA using GraphPad Prism4 software or iWorks Numbers.

## Results

### Induction of multinucleation is not shared by all chlamydial species

**Figure 1:**
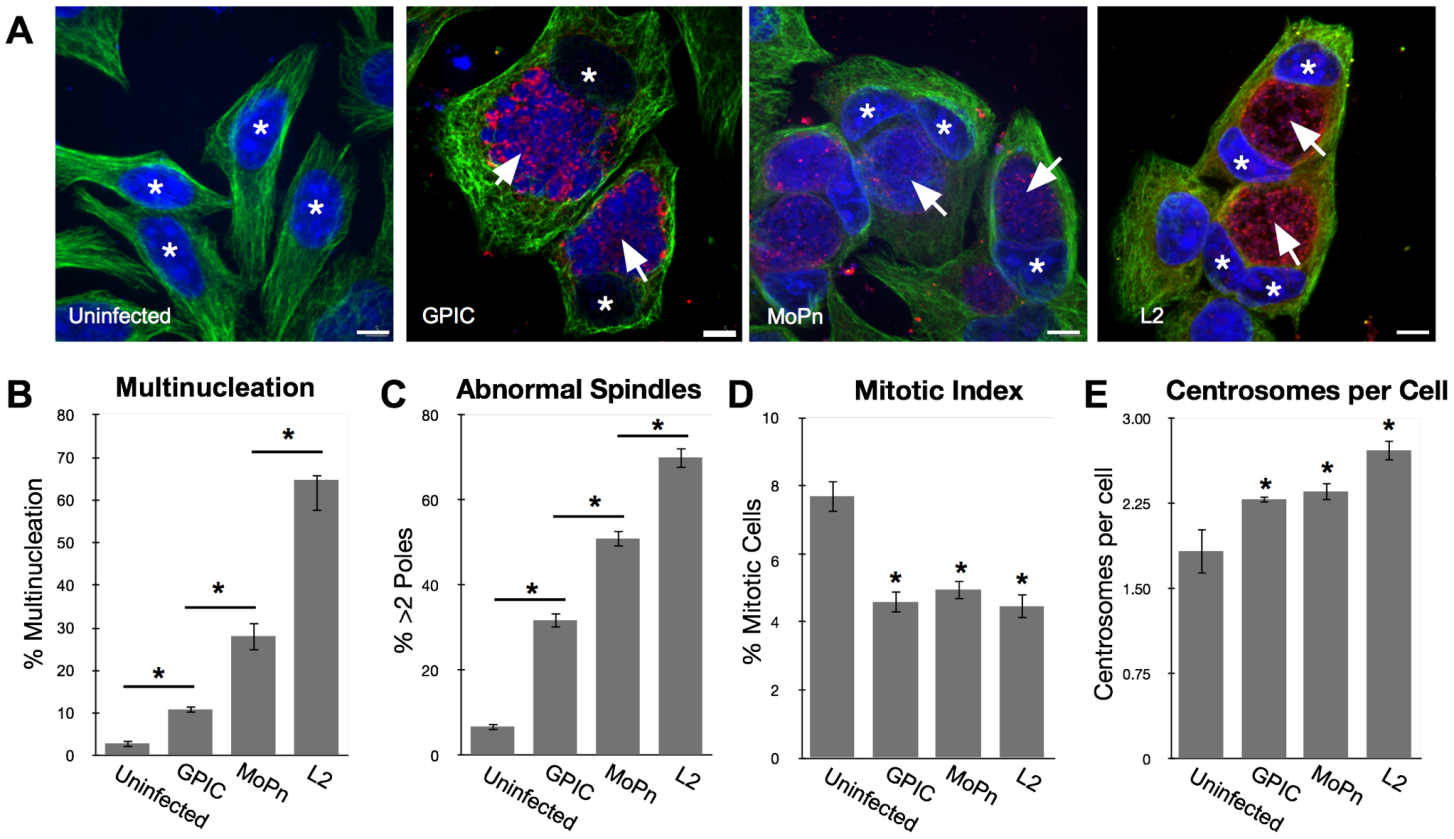
Multinucleation induction by different chlamydial species. [A] HeLa cells infected with *Chlamydia trachomatis* (L2), *C. muridarum* (MoPn) or *C. caviae* (GPIC) for 40 hours. Cells were stained with anti-β-tubulin (green), human serum (red), and Draq5 (blue) (representative cells shown, asterisks denote the nuclei and arrows indicate chlamydial inclusions). [B] Multinucleation induction for all three species was statistically higher than uninfected. Each species also induced a significantly different level of multinucleation from each other, p<0.01, N = 3 experiments with at least 600 cells per experiment. [C] The mitotic index was reduced in cell infected with all three chlamydial species. There was a statistically significant difference between the uninfected and GPIC-, MoPn- and L2- infected cells, (T-test p<0.01), N = 3 experiments, >1500 cells per experiment but no significant difference between species. [D] Centrosome amplification after infection with all three species was significant when compared to uninfected but not significant when compared between species (T-test p<0.01), N = 3 experiments, >150 cells per experiment. [E] The rates of induction of abnormal spindles was higher after infection with all three chlamydial species. Induction of spindle abnormalities also differed significantly between species. (T-test p<0.01) N = 3, >100 cells per experiment. Bar on images = 5 nm.

Previous studies by our lab and others have shown that *C. trachomatis* infection causes multinucleation of host cells [9], [10], [24]. In this study the multinucleation rate induced by the closely related species MoPn and the more divergent species GPIC was ascertained to determine if this phenotype was conserved throughout the *Chlamydia* genus [Figure 1]. Uninfected HeLa cells had a multinucleation rate of 2.9±1.0%. GPIC and MoPn infection caused multinucleation rates of 10.9±0.9% and 28.1±0.6% respectively at 40 hours post-infection (hpi), significantly lower than the 64.7±2.5% observed in Ctr L2-infected cells [Figure 1B]. Although we have shown that genomic instability is the major cause of multinucleation during a chlamydial infection [10], others report that steric interference of a large mature inclusion may contribute [8]. To rule out the effect of steric differences between inclusions from these different species, the area of each inclusion was measured at 40 hpi to compare inclusion sizes. The average inclusion size of GPIC, MoPn and L2 did not vary significantly (328.1±93.3 µm^2^, 360.3±122.2 µm^2^, and 350.6±139.4 µm^2^ respectively, p-value>0.3) and is therefore unlikely to contribute meaningfully to the different rates of multinucleation.

### Induction of multipolar spindles is not conserved between species

Our previous work demonstrated that multinucleation induced by chlamydial infection results from the induction of genomic instability [10]. *C. trachomatis* infection was shown to induce multipolar spindle formation during mitosis and that these defective spindles in turn caused chromosomal segregation errors that directly contributed to multinucleation. We therefore examined cells infected with the three distinct chlamydial species for their ability to induce multipolar spindles. The three species of *Chlamydia* induced significantly different rates of spindle pole defects. Ctr L2 caused mitotic spindle pole defects in 70.1±2.6% of mitotic cells, MoPn and GPIC only caused spindle pole defects in 51.0±2.0% and 31.8±1.8% of mitotic cells, respectively [Figure 1C]. The correlation observed in these strains between multinucleation and spindle pole defects supports the hypothesis that genomic instability caused by the induction of multipolar mitosis contributes to multinucleation.

### Infection with all three chlamydial species (GPIC, MoPn and Crt L2) caused a decrease in the mitotic index and induced centrosome amplification

*Chlamydia* infection causes multipolar spindle formation during mitosis through the induction of multiple phenotypes; infection causes dysregulation of centrosome number control, inhibition of centrosome clustering and early anaphase onset [10], [11], [25]. Therefore we determined whether infection with these three chlamydial species induced different effects on centrosome amplification and early mitotic exit.

To determine the effects of infection on mitosis, we measured the mitotic index in cells infected with C. trachomatis L2, MoPn and GPIC. The mitotic index is a measure of the fraction of cells in mitosis as compared to interphase. We have previously documented that *C. trachomatis* infection does not significantly change the time cells spend in interphase, but causes a dramatically shortened mitosis by causing premature anaphase onset indicating a loss of checkpoint control [11]. Therefore, the mitotic index yields a measure of premature mitotic exit and a reduction in spindle assembly checkpoint (SAC) control. As expected, Ctr L2- infected cells induced a significantly lower mitotic index than uninfected cells. Both MoPn and GPIC infection similarly resulted in a decrease in the mitotic index, with no significant difference between any of the species [Figure 1D].

We have also previously reported that Ctr L2 infection results in significant centrosome amplification defects and speculated that these defects contribute to multinucleation [25]. Centrosome numbers per cell were measured for Ctr L2, MoPn, and GPIC to compare centrosome duplication effects caused by infection. All three species caused centrosome amplification defects similar to that of Ctr L2, the levels of which did not vary significantly between cells infected with the different *Chlamydia* species [Figure 1E]. These data indicate that the observed differences in the induction of multinucleation caused by each species is not likely due to changes in mitotic exit or centrosome amplification.

### Rates of Centrosome mislocalization differ between species

The observation that of the three phenotypes associated with chlamydial induced multinucleation, only the induction of multipolar spindles correlated with the differential rates of multinucleation between species suggests that effects on centrosome clustering may differ during infection with these three species. Physical clustering of centrosomes is key to suppression of the effects of centrosome amplification and prevention of multipolar mitotic spindle persistence [11].

**Figure 2:**
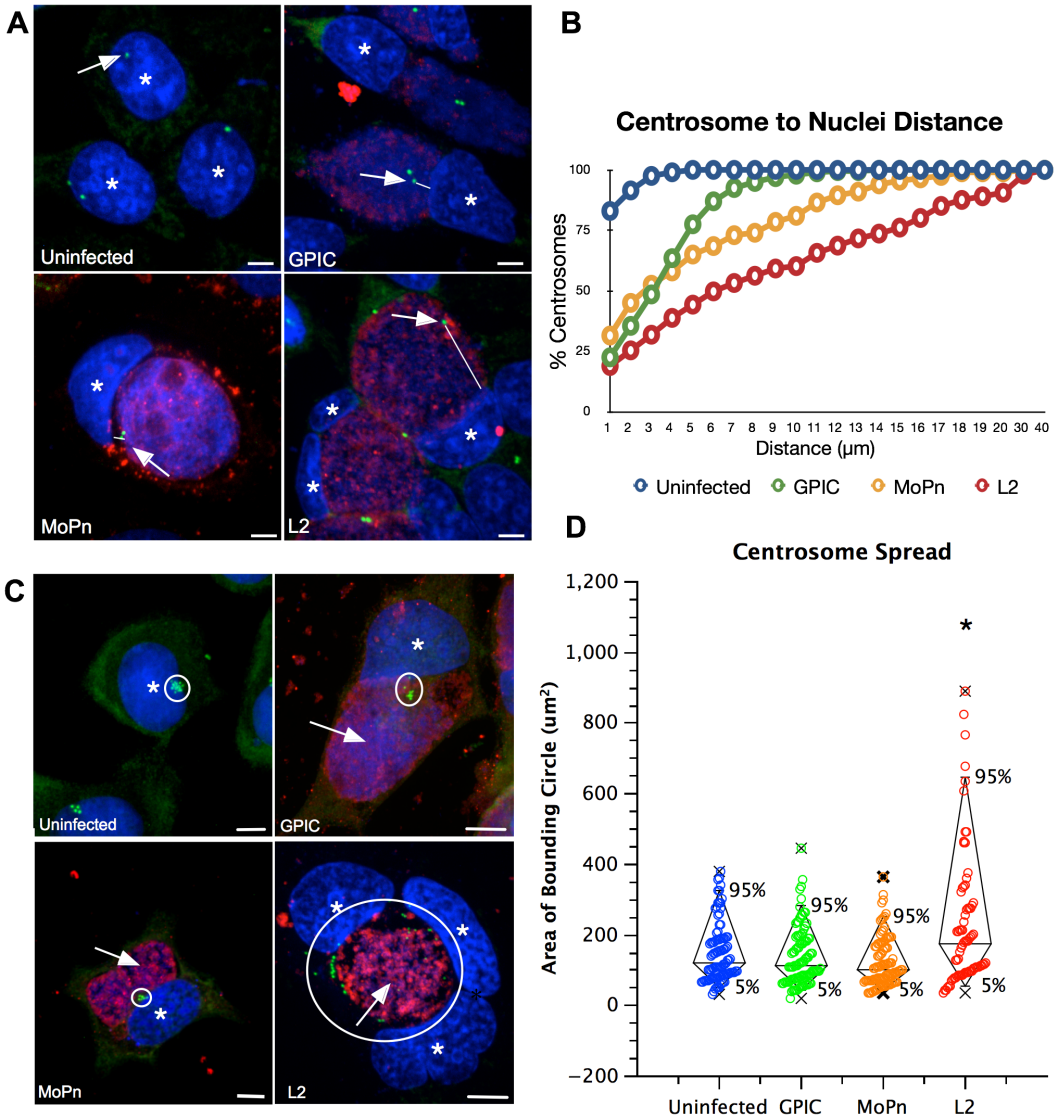
Centrosome positioning. [A] HeLa cells were infected with L2, GPIC, or MoPn for 40 hours. Cells were stained with anti-γ-tubulin (green), human serum (red), and Draq5 (blue). Arrows indicate centrosomes, asterisks identify nuclei, and the lines indicate example distances measured. [B] The distance between all centrosomes within the cell and the closest point in the nucleus was measured. Uninfected cells had 92% of the centrosomes within 2 µm of the nucleus. In infected cells at least 90% of the centrosomes where within 7 µm (GPIC), 13 µm (MoPn) and 20 µm (L2). There is a significant difference in the mean distance between populations (ANOVA p<0.01). N>200 cells. Bar on images = 5 nm. [C] Neuroblastoma cells infected with L2, MoPn or GPIC for 40 hours. Cells stained with anti-γ-tubulin (green), human serum (red), and Draq5 (blue) (asterisks identify cell nuclei and arrows indicate chlamydial inclusions). [D] The centrosome spread was measured with a bounding circle and graphed with a box and whisker plot (X denotes minimum and maximum, box encompises 5–95% interval). There was no significant difference between uninfected and GPIC- or MoPn-infected cells (ANOVA p>0.05), centrosome spread in Ctr L2-infected cells was significantly increased when compared to uninfected. (T-test p<0.01, N>100 cells). Bar on images = 5 µm.

Our data previously suggested that chlamydial induced centrosome positioning defects during interphase are a contributing factor to the induction of multipolar spindles leading to genomic instability [11]. To determine if the induction of centrosome localization defects differ between species, we measured both the centrosome to nucleus distance in HeLa cells infected with the different species of *Chlamydia* as well as centrosome spread in neuroblastoma cells which are defective in regulation of centrosome duplication [Figure 2]. As expected, >90% of the centrosomes were within 2 µm of the nucleus in uninfected Hela cells [Figure 2A and B]. However, centrosomes were partially mislocalized and positioned further from the nucleus in cells infected with GPIC, with >90% of the centrosomes located within 7 µm of the nucleus. MoPn-infected cells had a further increase in centrosome to nucleus distance with >90% of centrosomes within 13 µm of the nucleus and >90% of centrosomes were within 20 µm of the nucleus in Ctr L2-infected cells [Figure 2B]. This data suggests that the inclusions formed by the chlamydial species all interact with centrosomes but cause differing levels of localization defects.

The neuroblastoma cell line is defective in centrosome number control; each cell contains 8–10 centrosomes and is therefore a useful cell line to study centrosome organization. Centrosome spread was determined by measuring the area of a bounding circle that surrounds all the centrosomes in a cell [11]. Uninfected cells had centrosomes occupying an area of 147±8 µm^2^ [Figure 2C and D)]. The centrosome spread in cells infected with GPIC or MoPn was not significantly different than uninfected cells, 137±8 µm^2^ and 122±7 µm^2^ respectively. However, consistent with our previous work, the centrosome spread of neuroblastoma cells infected with Ctr L2 increased to an average of 226±22 µm^2^, significantly different from uninfected cells and cells infected with the other two species (p<0.01, Figure 2C and D). Together these data suggest that the three species of chlamydia interact differently with the host centrosomes and microtubules and therefore induce varying levels of centrosome positioning errors.

### Inhibition of centrosome clustering in GPIC-infected cells causes multipolar spindles and multinucleation

**Figure 3:**
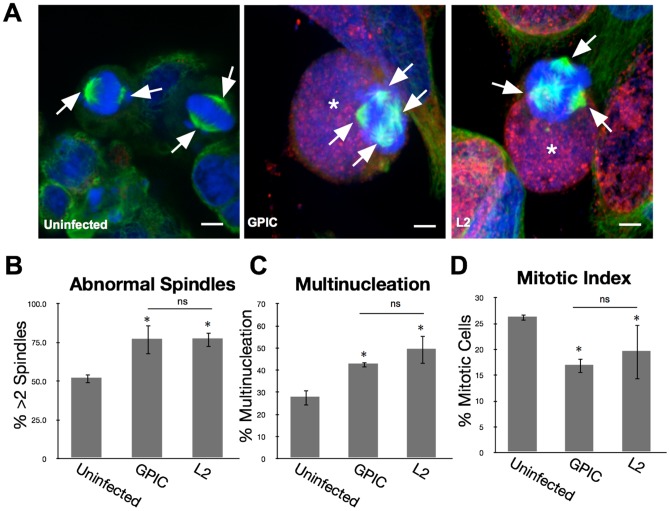
Declustering of centrosomes by griseofulvin. [A] HeLa cells infected with L2 or GPIC for 24 hours and treated with 20 µM griseofulvin for a further 16 hours. Cells stained with anti-β-tubulin (green), human serum (red), and Draq5 (blue). Arrows denote spindle poles and the asterisks denote the nuclei. [B] Percent of mitotic cells that contained more than 2 spindle poles were evaluated. When treated with griseofulvin the GPIC infected cells had the percentage of mitotic cells with multipolar spindles increase significantly when compared to uninfected (T-test p<0.01). This increase matched that of L2 induced multipolar spindle rate as there was no significant difference between the two. [C] Multinucleation of infected cells treated with griseofulvin was evaluated. Griseofulvin treatment led to an significant increase in multinucleation for GPIC infected cells compared to uninfected (T-test p<0.01). The level of multinucleation in the GPIC infected, griseofulvin treated cells matched that of L2 infected and griseofulvin treated cells as there was no significant difference between the two. [D] Mitotic index measurements were performed on griseofulvin treated cells. The mitotic index of GPIC and L2 infected cells treated with griseofulvin was significantly decreased for both chlamydial species (ANOVA p<0.01). There was no significant difference in the mitotic index between chlamydial species (T-test p>0.05). N = 3 experiments, >1500 cells per experiment. Bar on images = 5 nm.

Both centrosome amplification and inhibition of centrosome clustering are required for induction of multipolar spindles [11]. Therefore, we reasoned that if centrosome clustering in GPIC-infected cells was inhibited, the rate of multipolar spindles would increase. To inhibit centrosome clustering, infected cells were treated with 10 µM griseofulvin. Griseofulvin is an antifungal compound that inhibits centrosome clustering in cancer cells [21]–[23]. Uninfected cells treated with griseofulvin had greater than two spindles poles in 51±3% of mitotic cells. Cells infected with Ctr L2 and treated with griseofulvin had a multipolarity rate of 77±5%. In GPIC-infected cells, griseofulvin treatment increased the frequency of multipolar spindles from 32% [see Figure 1C] to 77±9%, similar to that of Ctr L2-infected cells, indicating that multipolar spindles could be induced in GPIC-infected cells when centrosome clustering was inhibited [Figure 3A and B].

Multinucleation during chlamydial infections correlates with induction of multipolar spindles, therefore we measured the effect of griseofulvin on the induction of multinucleation. When centrosome clustering was inhibited in GPIC-infected cells, multinucleation increased from 11±1% to 43±1% [Figure 3C]. This increase matched the multinucleation rate of 49±6% in griseofulvin-treated Ctr L2-infected cells, demonstrating that GPIC infection could induce multinucleation to levels similar to Ctr L2 when centrosome clustering was inhibited.

As griseofulvin interferes with microtubule function, we would expect it to activate the spindle assembly checkpoint (SAC) and cause cells to arrest in mitosis. Therefore, we again measured the mitotic index (a measure of time cells are in mitosis) in these cells. The mitotic index for uninfected, griseofulvin-treated cells was 26±1% which decreased to 19±4% for Ctr L2-infected cells and 17±1% for GPIC-infected cells [Figure 3D]. This decrease was statistically different from uninfected for both Ctr L2 and GPIC, lending additional support to the observation that both Ctr L2 and GPIC override the SAC.

### Induction of DNA segregation errors is not conserved between chlamydial species

**Figure 4:**
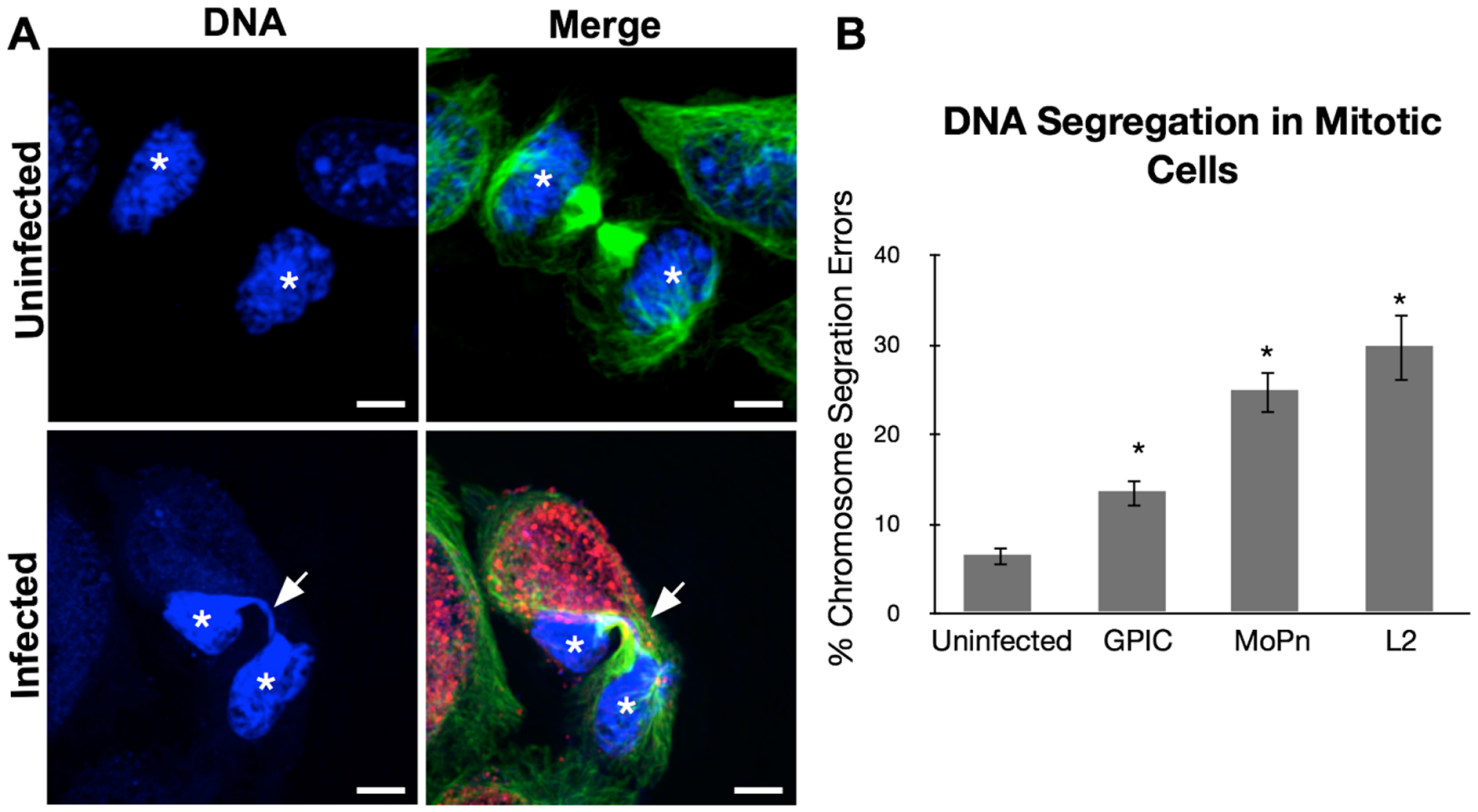
DNA segregation errors correlate with multinucleation. [A] HeLa cells infected with L2, MoPn or GPIC for 40 hours. Cells were stained with anti-β-tubulin (green), human serum (red), and Draq5 (blue). Nuclei are noted with asterisks and DNA bridging is identified with the arrow. [B] Cells exiting mitosis were evaluated for DNA bridges and lagging chromosomes. L2 infected cells show the largest increase in DNA segregation errors while GPIC infectected cells show the least. Infection with any of the three species lead to significantly higher rates of DNA segregation errors from uninfected (T-test, p<0.01) N = 3 experiments with ≥100 cells per experiment. Rates of DNA segregation errors induced by each species also varied significantly from one another (T-test, p<0.01). Bar on images = 5 nm.

A primary driving mechanism leading to multinucleation in *Chlamydia*-infected cells is the induction of DNA segregation errors leading to DNA bridging between daughter cells [10]. Therefore, we scored uninfected and infected HeLa cells exiting mitosis for DNA bridges. In uninfected cells, DNA bridges were present in 6±1% of cells that exited mitosis [Figure 4]. This number increased to 14±2% in GPIC-infected cells, 25±3% in MoPn-infected cells and 30±4% in Ctr L2-infected cells [Figure 4]. MoPn- and Ctr L2- infected cells demonstrated a significant increase of DNA bridging over uninfected cells. The pattern of rates of DNA segregation errors correlated with both induction of multipolar spindles as well as the induction of multinucleation across species (see Figure 1B and C), further supporting the observation that DNA bridging is an important factor in causing multinucleation.

### The conserved chlamydial protease CPAF contributes to the induction of multinucleation

Taken together, our data indicates that induction of multinucleation was not completely conserved across the chlamydial species and that this difference was linked to the inhibition of centrosome clustering and induction of DNA segregation errors. Interestingly, all the chlamydial species tested induced supernumerary centrosomes and a decreased mitotic index; phenotypes that we hypothesize are critical to the induction of multinucleation. We have previously shown that the conserved chlamydial protease CPAF is capable of degrading two important proteins regulating anaphase onset, thereby controlling mitotic exit [10]. We hypothesised that the CPAF protease would be essential for the induction of multinucleation though its influence on mitotic exit control. To directly test the role of CPAF in centrosome amplification, early mitotic exit and induction of multinucleation, two CPAF mutants (rst17 and M532), a wt CPAF strain (rst5), and a GspE mutant defective in Type II secretion were assayed. The M532 mutant contains nine non synonymous mutations that differ from the parent wt L2 including one that creates an early stop codon in the CPAF gene rendering the mutant incapable of expressing an active CPAF protein [Figure S1]. The mutation changes the glutamine at position 44 to a stop codon leading to a severely truncated protein containing no catalytic domains. The rst17 and the wt *cpa* isogenic strain rst5, were derived from the M169 mutant, an independent CPAF mutant isolated from the same library. The rst17 mutation changes the tryptophan at 294 to a stop leading to a truncated ORF that codes for the n-terminal half of the CPAF protein but not the c-terminal half. Both halves are required for catalytic activity as CPAF is extensively modified by self cleavage and assembles into a final active form consisting of the two peptides [26]. The rst5 and rst17 mutants share all but two non synonymous genetic changes (*cpa* and CTL0884) the *cpa* locus codes for the CPAF protein [Figure S1]. The isolation and characterization of these mutants will be described in full detail in the context of a comprehensive study focusing on the pathogenesis of the CPAF mutants (Snavely, Kokes, Nguyen et al. submitted). Importantly, the only non synonymous change in common between the two CPAF null clones, rst17 and M532, that isn't also present in the CPAF positive rst5 clone is in the *cpa* locus as the S555 to F555 amino acid change in CTL0884 is not present in the M532 mutant. This allows us to have high confidence that differences in phenotypes between these clones can be attributed to the *cpa* locus. These strains have been completely sequenced and the genotypes of each mutant is listed in Figure S1. CPAF is a protease that is secreted from the *Chlamydia* RBs as an inactive zymogen in a Sec dependent process [27]. Therefore, we also investigated multinucleation in a GspE chlamydial mutant. The GspE mutant isolate is defective in Type II secretion and is incapable of secreting the CPAF protein from the chlamydial cytoplasm [20].

**Figure 5:**
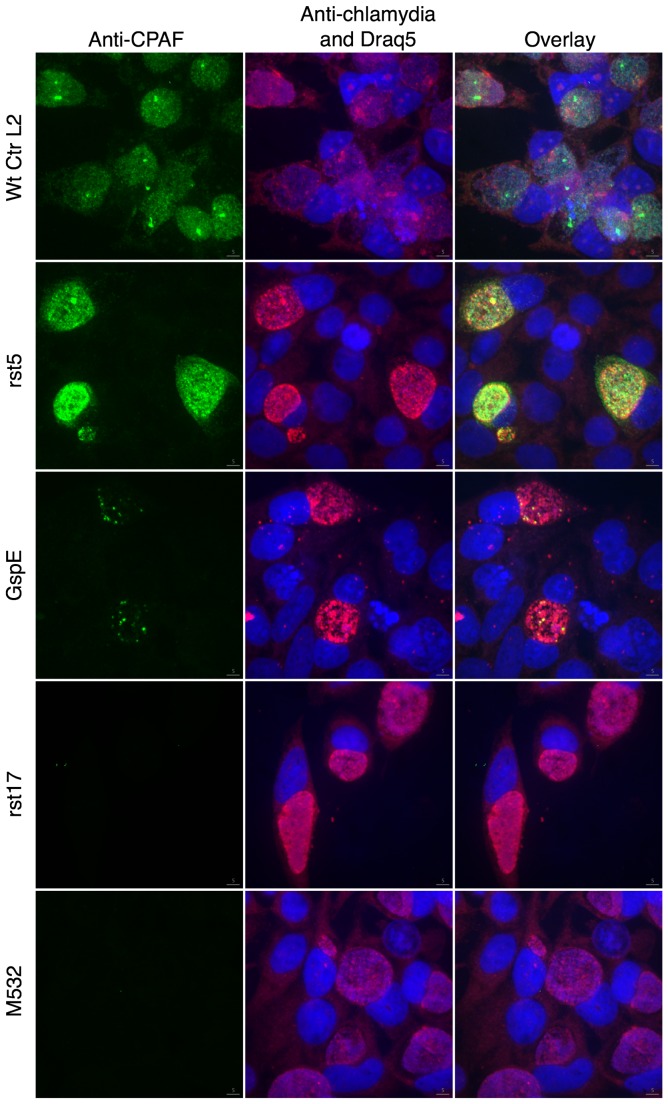
Immunofluorescent localization of CPAF. HeLa cells infected with C. trachomatis L2, or mutant isolates rst5, rst17, M532, GspE for 40-CPAF antibody (green), Anti-Chlamydia antibody (red), DNA (Draq5).

To verify that these mutants did not express CPAF, we infected HeLa cells with each mutant and stained for the CPAF protein using IFA [Figure 5]. Fluorescent confocal microscopy verified that the rst17 and M532 clones did not express any detectable CPAF protein, while both wt Ctr L2 and rst5 strains had detectable CPAF staining in the bacteria, inclusion and cytoplasm of the host cell [Figure 5]. Staining for the CPAF protein in the GspE mutant revealed staining only in a subset of bacteria and no signal in the inclusion or host cytoplasm [Figure 5]. This staining pattern suggests the GspE mutant did not secrete CPAF across the bacterial outer membrane.

**Figure 6:**
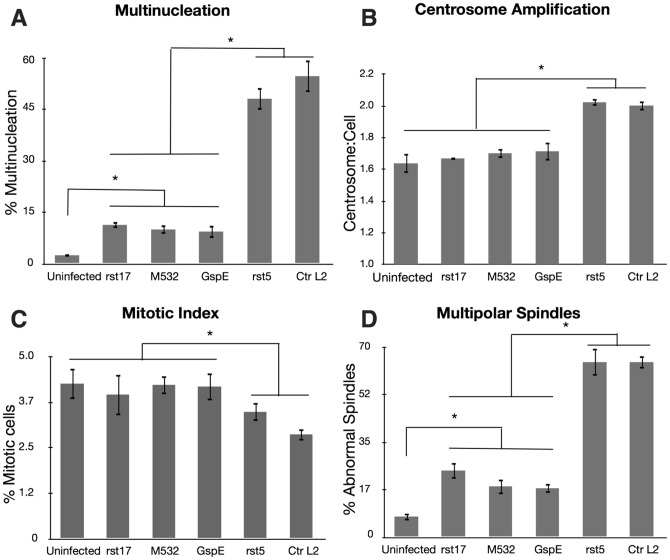
Multinucleation is CPAF dependent. HeLa cells infected with C. trachomatis L2, or mutant isolates rst5, rst17, M532, GspE for 40[A] Multinucleation induction by the CPAF mutants was significantly lower than the CPAF positive isolates, (ANOVA p<0.01). N = 3 experiments, >600 cells per experiment. However, the CPAF mutants still induced significantly higher rates of multinuclation than uninfected cells. (ANOVA p<0.01). N = 3 experiments, >600 cells per experiment. [B] Centrosome amplification in the CPAF deficient strains differed significantly from the CPAF wt strains but were not statistically different from uninfected, (ANOVA p<0.01). N = 3 experiments, >150 cells per experiment. [C] The mitotic index of cells infected with the CPAF deficient strains was not significantly different from uninfected cells. However, the CPAF positive strains reduced the mitotic index significantly compared to uninfected, (ANOVA p<0.01). N = 3 experiments, >800 cells per experiment. [D] Multipolar spindle induction was significantly higher in the CPAF wt strains than CPAF null isolates. However the CPAF deficient strains still showed a small but significantly higher rate than uninfected, (ANOVA p<0.01). N = 3 experiments, >100 cells per experiment.

The chlamydial isolates containing CPAF mutations caused significantly less multinucleation than strains with an intact *cpa* coding sequence. Cells infected with rst17 and M532 caused multinucleation in 11±1 and 10±1% of cells respectively [Figure 6A]. Comparatively, infection with wt Ctr L2 and the wt cpa isogenic strain, rst5, resulted in multinucleation in 54±5 and 48±3% cells respectively [Figure 6A]. The GspE secretion mutant also did not induce high levels of multinucleation with a rate of 9±2% multinucleated cells. Although there was a dramatic decrease in multinucleation between the *cpa* mutants and the wild type strains there was still a small but statistically significant increase in multinucleation in cells infected with all *cpa* mutants over uninfected [Figure 6A].

### CPAF is required for both centrosome amplification and reduced mitotic index

We next looked at two of the phenotypes determined to be associated with chlamydial induction of multinucleation. We infected HeLa cells with the different mutants and measured centrosome amplification and mitotic index. To quantitate effects on centrosome numbers HeLa cells were infected, fixed and stained for γ-tubulin. None of the *cpa* mutants induced centrosome amplification, 1.7±0.01 centrosome/cell for rst17 and 1.7±0.02 centrosome/cell for M532 compared to 1.6±0.06 centrosome/cell for uninfected cells. The rst5 mutant and wild type Ctr L2 caused significant centrosome amplification with rst5-infected cells having 2.0±0.02 centrosome/cell and 2.0±0.03 centrosomes/cell for wt Ctr L2 [Figure 6B]. The GspE secretion mutant was also tested for its effects on centrosome number control. Like the *cpa* mutants, the GspE mutant strain did not cause significant centrosome number changes compared to uninfected [Figure 6B].

To determine whether the CPAF mutation impacted the effect of infection on the length of mitosis, cells were infected with WT and the CPAF mutants, fixed and stained for DNA at 36 hours post infection. We counted the percentage of cells in mitosis to calculate the mitotic index [Figure 6C]. The *cpa* mutants lost the ability to reduce the mitotic index in infected cells having a mitotic index similar to that of uninfected cells. Conversely, rst5 and wt Ctr L2, strains which express CPAF, reduced the mitotic index significantly. Again, the phenotype of the GspE mutant was similar to the *cpa* null mutants with no significant reduction in the mitotic index.

The average inclusion size for all the strains tested was measured to discount differences in steric effects between the inclusions of the different mutant isolates. Using the image data set from above we found that the inclusions of all strains were comparable in size [Table 1].

**Table 1:** Inclusion size 40

Isolate	Inclusion area µm^2^
**Ctr L2**	16±3
**rst5**	20±5
**rst17**	19±4
**M532**	21±6
**Gspe**	15±4

### CPAF is required for the induction of multipolar spindles but not centrosome positioning defects

As we hypothesize that the induction of multipolar spindles requires both centrosome amplification and inhibition of centrosome clustering, we next measured the induction of multipolar spindles and quantified centrosome positioning defects. The CPAF mutant strains rst17 and M532 along with the GspE mutant all caused significantly less multipolar spindles in mitotic cells (24±3%, 17±3%, and 18±2% respectively) than the CPAF wt strains rst5 and wt Ctr L2, (64±5% and 64±3%) [Figure 6D]. However, similar to the multinucleation data, the CPAF mutants had a small but statistically significant increase in multipolar spindle formation when compared to uninfected cells (8±2%) [Figure 6D].

**Figure 7:**
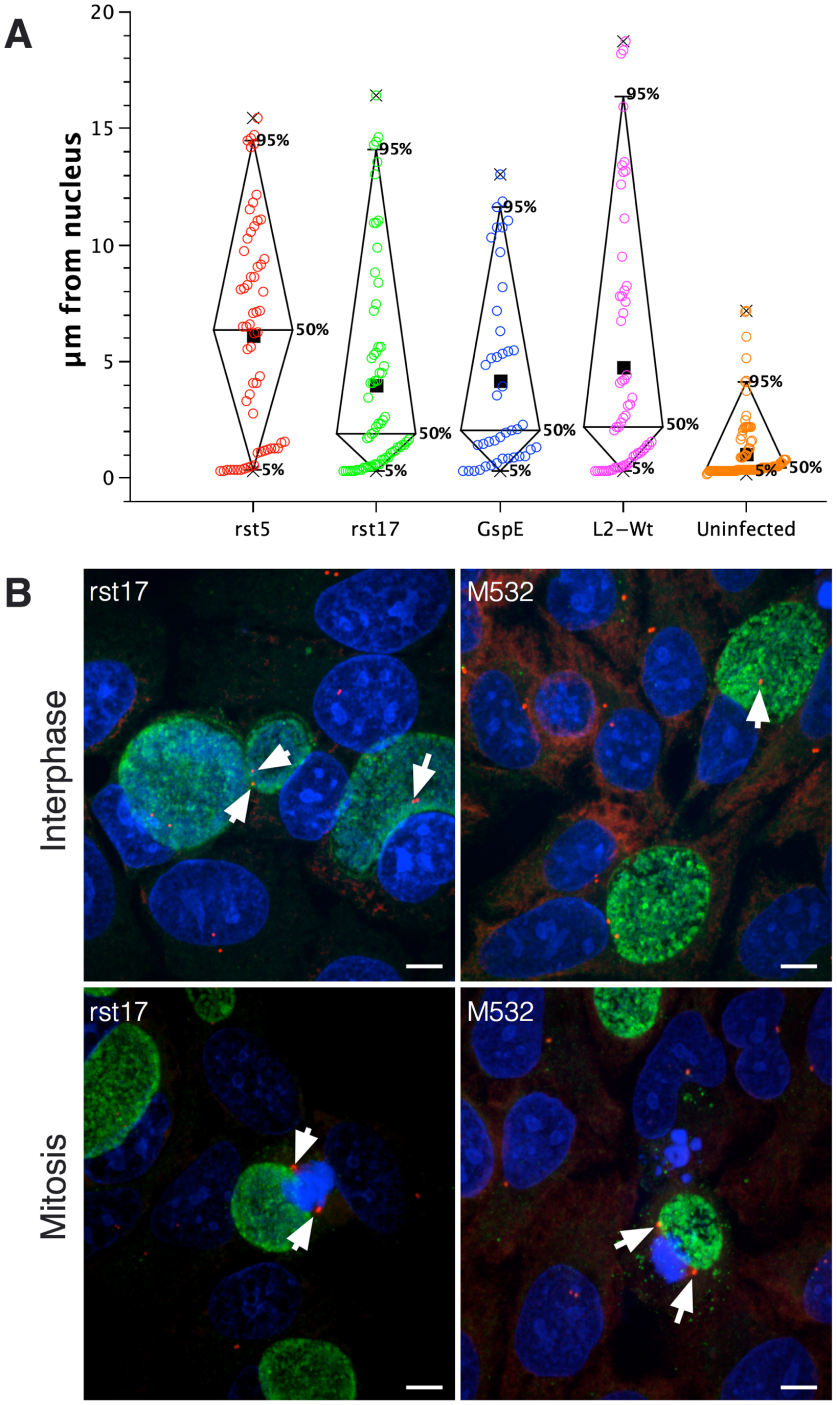
CPAF mutants continue to disrupt centrosome positioning and associate with spindle poles. HeLa cells were infected with the *cpa* mutant rst17, GspE, isogenic strain rst5 and wt L2 for 40 hours. [A] The distance between the centrosomes and the closest point in the nucleus was measured and graphed. The centrosomes in cells infected with all chlamydial mutant strains and wt L2 were positioned at significantly greater distances from the nucleus than uninfected cells (ANOVA p<0.01, N>100 centrosomes). Minimum and maximum are denoted with X and the boxes represent the population mean. There was no significant difference in centrosome positioning between the mutant strains ANOVA p>0.05, N>100 centrosomes. [B] Representative images of cells infected with the CPAF mutants rst17 and M532. Cells stained with anti-γ-tubulin (red), human serum (green), and Draq5 (blue). Arrows point to the centrosomes associated with the chlamydial inclusions in both interphase cells as well as mitotic cells. Bar on images = 5 µm.

We next determined the effect of the CPAF mutation on centrosome positioning. Centrosomes are tightly bound to the chlamydial inclusion, causing the centrosomes to reposition from a perinuclear location to the chlamydial inclusion [11], [25]. The *cpa* mutant rst17, the GspE secretion mutant, rst5 strains and wt Ctr L2 all caused defects in centrosome positioning when compared to uninfected cells [Figure 7A]. Additionally, we assayed for association of the CPAF mutants inclusions with centrosomes and spindle poles. Both the rst17 and M532 *cpa* mutant strains associated with centrosomes during interphase and the spindle poles during mitosis [Figure 7B].

### Rescue of multinucleation

**Figure 8:**
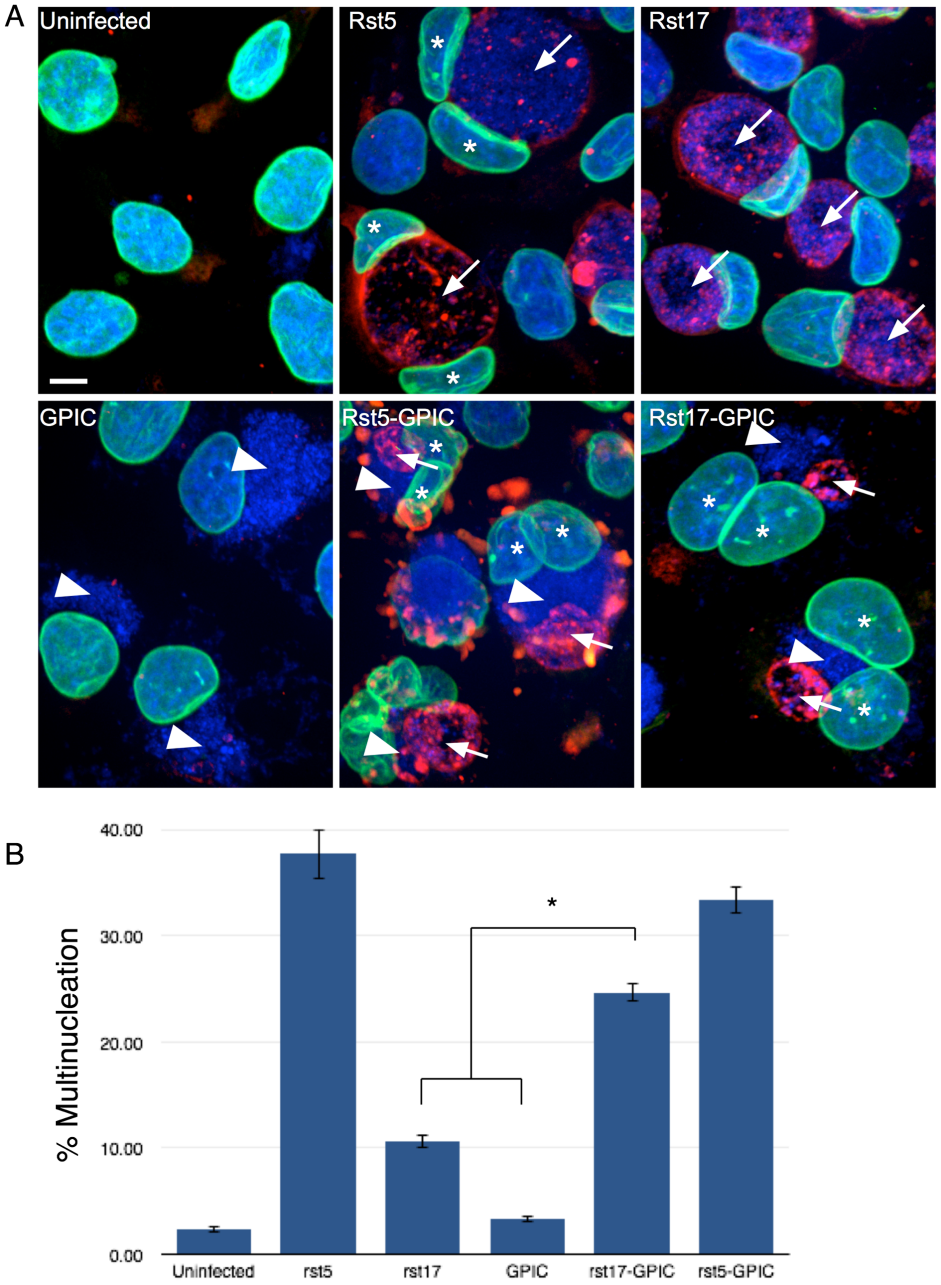
Rescue of multinucleation. [A] HeLa cells infected with GPIC, rst5, rst17 or co-infected with GPIC-rst5 and GPIC-rst17 for 40 hours. Cells were stained for laminA/C (green), C. trachomatis (red) and for DNA (blue). Arrows indicate *C. trachomatis* inclusions and arrowheads indicate GPIC inclusions. Stars highlight the nuclei in multinucleated cells. Bar on images = 5 µm. [B] Multinucleation was quantified for each infection, 3 experiments n>100 cells each experiments. Co-infection with rst17 and GPIC rescued multinucleation when compared to rst17 and GPIC alone. *  = T-test p value<0.01.

Our data demonstrates that, in contrast to wild type *Chlamydia*, CPAF null mutants lack the ability to induce significant multinucleation. CPAF null mutants do not induce centrosome amplification or cause a reduction in the mitotic index of infected cells, two phenotypes that we propose are critical for the induction of high levels of multinucleation during chlamydial infection. However, the inclusions of CPAF mutants retained the ability to disrupt centrosome positioning. Infection with GPIC also did not induce high levels of multinucleation, although GPIC infected cells had significant centrosome amplification and caused a reduction in the mitotic index. In contrast to CPAF null mutants, GPIC infection did not cause significant centrosome positioning errors during infection. Therefore, we asked if multinucleation could be restored by coinfection with GPIC and rst17, providing the effects of centrosome amplification from GPIC and centrosome declustering through infection with the CPAF mutant. HeLa cells were infected with rst5 (*cpa* positive control), rst17, and GPIC for controls and co-infected with rst17-GPIC and rst5-GPIC [Figure 8]. As expected, infection with rst5 (*cpa* wt) resulted in high levels of multinucleation (∼38%), while infection with rst17 (*cpa* null) and GPIC caused only moderate levels of multinucleation (∼10% and ∼3% respectively) [Figure 8]. Co-infection of cells with GPIC and rst17 restored multinucleation to levels similar to rst5 (∼25%), while co-infection of cells with rst5-GPIC did not substantially change multinucleation (∼33%) [Figure 8]. This data supports the role of two effector pathways in the induction of multinucleation and demonstrates that these two pathways are not linked as they can be provided from separate organisms during infection.

## Discussion

We had previously shown that *C. trachomatis* L2 infection causes multinucleation through cytokinesis failure induced by DNA bridging between daughter cells [10]. This observation led us to hypothesize that induction of DNA segregation errors is the driving force causing multinucleation. We further demonstrated that DNA segregation errors are due to a complex interaction between three infection induced phenotypes; centrosome amplification, centrosome positioning errors, and relaxation of the SAC mitotic checkpoint [10], [11]. In this study we demonstrated induction of multinucleation is not completely conserved among *Chlamydia* spp. Infection with Ctr L2 led to very high levels of multinucleation while GPIC infection caused only a modest increase. MoPn-infected cells had an intermediate phenotype inducing multinucleation at levels more similar to Ctr L2 infection. Interestingly infection with all three species led to early mitotic exit (as measured by reduced mitotic index) and caused amplification of centrosomes to similar levels. However, GPIC infection caused considerably less centrosome positioning errors during interphase and mitosis than did the other two species. These data suggest that the variation between the interaction of different chlamydial species' inclusions with centrosomes contributes to the differences in induction of multinucleation. Additional evidence for this hypothesis is that GPIC-infected cells were induced to cause high levels of multinucleation and spindle pole defects when centrosome clustering was inhibited with griseofulvin. This observation lends further support to the hypothesis that the differences in multinucleation induction between Ctr L2 and GPIC infection is due to differences in the inclusions interaction with dynein and the microtubule network.

The observation that centrosome amplification and reduced mitotic index are conserved while centrosome positioning defects are not, suggests that these three phenotypes are induced by at least two different chlamydial effector pathways; one pathway that is highly conserved and one that is species specific. Mital et al. demonstrated that inclusion microdomains, which include Inc proteins, are closely associated with centrosomes and that these microdomains differ in composition between chlamydial species [28]. Additionally, chlamydial inclusion membrane proteins (Incs) are poorly conserved across chlamydial species, [29] suggesting that Inc protein interactions with centrosomes could account for the species specific effector pathway leading to multinucleation.

We had previously hypothesized that the chlamydial protease CPAF is involved in the multinucleation phenotype [10]. CPAF is a chlamydial protease that is secreted from the RB in a type II dependent manner [27]. The CPAF enzyme is highly conserved in all pathogenic *Chlamydia* making it a candidate for the conserved factor involved in multinucleation. The data presented in this paper show that there is a dramatic decrease in multinucleated cells during infection in *C. trachomatis* L2 strains that do not express or secrete CPAF. Additionally, chlamydial isolates deficient in CPAF activity did not induce centrosome number defects in infected cells and induced less multipolar spindles as well. These data demonstrate that CPAF contributes significantly to the induction of multinucleation, through the induction of centrosome number defects and effects on mitotic control. The mechanism through which CPAF operates to cause such disparate phenotypes as centrosome amplification and early mitotic exit are not yet known. We speculate that its action is likely direct as CPAF secretion is required for this activity. However, it is possible that CPAF may be involved in the maturation of a secondary chlamydial factor, either by direct activation or by facilitating secretion into the host cytoplasm. Careful dissection of the location and timing of CPAF's activity will be required to identify the specific targets cleaved for the induction of these phenotypes.

Interestingly, although multinucleation was drastically decreased in the CPAF null strains it was still higher than uninfected cells suggesting centrosome amplification and early mitotic exit are not absolutely required. Sun et al. demonstrated that steric effects of the chlamydial inclusion correlated with multinucleation [8]. Our data show that multinucleation is dependent on centrosome positioning defects, suggesting that the association between the chlamydial inclusion and the centrosomes can cause multinucleation at a low level without centrosome amplification, perhaps by causing steric interference with the centrosomes and the spindle apparatus during cell division.

**Figure 9:**
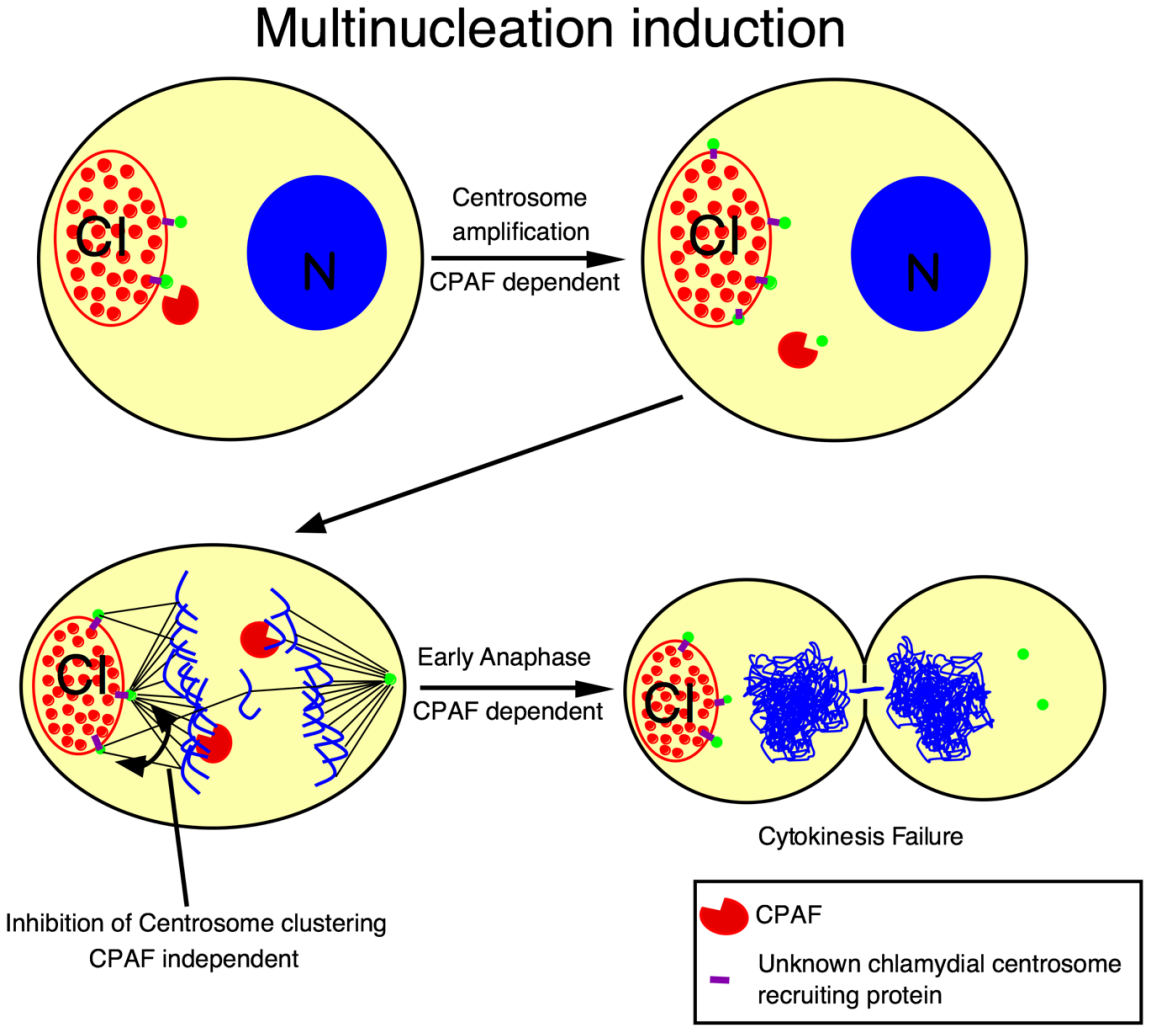
Model. Proposed model for the induction of genomic instability and multinucleation during chlamydial infection. Chlamydial inclusion (CI), host cell nucleus (N).

Together these data suggest that two independent effector pathways are involved in the induction of multinucleation during *C. trachomatis* L2 infection. One, CPAF dependent leading to centrosome amplification and early mitotic exit and a second pathway mediating minus-end microtubule interactions, likely mediated through binding to the dynein motor protein. This model has been diagramed in Figure 9. Further evidence that two independent pathways both contribute to multinucleation is the result showing that co-infection of cells with GPIC (centrosome amplification competent, low multinucleation) and a CPAF null C. trachomatis L2 (high centrosome interactions, low multinucleation) resulted in a rescue of multinucleation induction, suggesting that the two effector pathways induce multinucleation synergistically and can be contributed by two separate organisms.

*C. trachomatis* infections have been epidemiologically linked to cervical cancer in a number of studies [4], [5], [30]–[42]. Centrosome amplification and genomic instability are both linked to cancer progression and tumor severity in a number of different human cancers [14], [15], [43]. We demonstrate here that the observed genomic instability in chlamydial infected cells is caused by the combined effects of centrosome amplification, early mitotic exit and centrosome positioning errors and that all three are required for the induction of high levels of genomic instability and multinucleation. In addition, our data suggests that two chlamydial effector pathways are involved in inducing chromosome instability, providing a potential molecular mechanism connecting chlamydial infection with reproductive cancers.

## Supporting Information

Figure S1 CPAF mutant genotypes. [A] Nucleotide polymorphisms revealed after whole genome sequence analysis for the isolated mutants. [B] Schematic of the early stop codons in the M532 and rst17 mutants.
(PDF)
