# Supplementary material for: Multinucleation during C. trachomatis Infections Is Caused by the Contribution of Two Effector Pathways
Source: PLoS One. 2014 Jun 23;9(6):e100763. doi: 10.1371/journal.pone.0100763 (PMC4067387; doi:10.1371/journal.pone.0100763)
Supplement: Figure S1 — CPAF mutant genotypes. [A] Nucleotide polymorphisms revealed after whole genome sequence analysis for the isolated mutants. [B] Schematic of the early stop codons in the M532 and rst17 mutants. (PDF) [file pone.0100763.s001.pdf]

A

| Clone M532 | | | | | | |
|---|---|---|---|---|---|---|
| Location | Amino Acid Change | Change | Codon Change | Protein Effect | gene | locus_tag |
| 123,163 | | C -> A | | | | |
| 127,338 | | T -> A | CCT -> CCA | None | | CTL0103 |
| 141,972 | S -> F | G -> A | TCC -> TTC | Substitution | | CTL0111 |
| 144,164 | E -> K | G -> A | GAG -> AAG | Substitution | | CTL0113 |
| **289,759** | **Q -> Stop** | **C -> T** | **CAG -> TAG (amber)** | **Truncation** | **cpa** | **CTL0233** |
| 374,717 | G -> S | G -> A | GGT -> AGT | Substitution | | CTL0303 |
| 391,909 | | C -> T | CTC -> CTT | None | flhA | CTL0316 |
| 435,453 | A -> V | G -> A | GCT -> GTT | Substitution | nusA | CTL0352 |
| 448,523 | A -> V | G -> A | GCT -> GTT | Substitution | | CTL0364 |
| 493,667 | A -> V | G -> A | GCT -> GTT | Substitution | mhpA | CTL0403 |
| 565,977 | | C -> T | GTC -> GTT | None | surE | CTL0470 |
| 570,606 | | G -> A | | | | |
| 601,361 | G -> E | C -> T | GGG -> GAG | Substitution | oxaA | CTL0503 |
| 616,827 | | C -> T | GAG -> GAA | None | | CTL0518 |
| 631,979 | G -> S | C -> T | GGT -> AGT | Substitution | | CTL0535 |
| 637,039 | R -> C | C -> T | CGC -> TGC | Substitution | clpC | CTL0538 |
| 638,353 | F -> L | T -> C | TTT -> CTT | Substitution | clpC | CTL0538 |
| 666,801 | | G -> A | AAG -> AAA | None | | CTL0563 |
| 675,519 | H -> Y | G -> A | CAC -> TAC | Substitution | rpoB | CTL0567 |
| 693,265 | S -> N | G -> A | AGC -> AAC | Substitution | pykF | CTL0586 |
| 798,820 | G -> E | G -> A | GGA -> GAA | Substitution | pmpC | CTL0671 |
| 867,379 | | C -> T | TTC -> TTT | None | pheT | CTL0736 |
| 928,436 | E -> K | C -> T | GAA -> AAA | Substitution | hisS | CTL0805 |
| 929,717 | | C -> A | | | | |
| 943,453 | | G -> A | GGG -> GGA | None | | CTL0818 |
| 949,091 | | G -> A | GGG -> GGA | None | lipA | CTL0821 |
| 998,156 | T -> M | C -> T | ACG -> ATG | Substitution | tolB | CTL0862 |
| 1,014,167 | D -> N | C -> T | GAT -> AAT | Substitution | rpoD | CTL0879 |
| **Clone rst17** | | | | | | |
| 123,086 | | G -> A | | | | |
| 123,163 | | C -> A | | | | |
| 127,338 | | T -> A | CCT -> CCA | None | | CTL0103 |
| 135,167 | | C -> T | | | | |
| 157,211 | | C -> T | | | | |
| **290,514** | **W -> Stop** | **G -> A** | **TGG -> TGA (opal)** | **Truncation** | **cpa** | **CTL0233** |
| 456,892 | G -> R | G -> A | GGG -> AGG | Substitution | | CTL0369 |
| 763,691 | T -> I | C -> T | ACC -> ATC | Substitution | dapL | CTL0646 |
| 798,820 | G -> E | G -> A | GGA -> GAA | Substitution | pmpC | CTL0671 |
| 863,064 | | G -> A | GGG -> GGA | None | | CTL0729 |
| 869,346 | G -> E | G -> A | GGA -> GAA | Substitution | pheT | CTL0736 |
| 929,717 | | C -> A | | | | |
| **1,021,992** | **S -> F** | **C -> T** | **TCC -> TTC** | **Substitution** | | **CTL0884** |
| **Clone rst5** | | | | | | |
| 123,086 | | G -> A | | | | |
| 123,163 | | C -> A | | | | |
| 127,338 | | T -> A | CCT -> CCA | None | | CTL0103 |
| 135,167 | | C -> T | | | | |
| 157,211 | | C -> T | | | | |
| 270,665 | L -> F | C -> T | CTC -> TTC | Substitution | ftsH | CTL0213 |
| 456,892 | G -> R | G -> A | GGG -> AGG | Substitution | | CTL0369 |
| 630,944 | | G -> T | CTG -> CTT | None | nqrE | CTL0533 |
| 763,691 | T -> I | C -> T | ACC -> ATC | Substitution | dapL | CTL0646 |
| 798,820 | G -> E | G -> A | GGA -> GAA | Substitution | pmpC | CTL0671 |
| 863,064 | | G -> A | GGG -> GGA | None | | CTL0729 |
| 869,346 | G -> E | G -> A | GGA -> GAA | Substitution | pheT | CTL0736 |
| 929,717 | | C -> A | | | | |

B

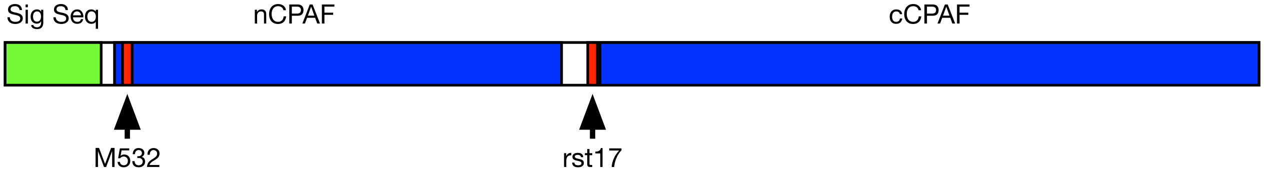


Supplemental Figure 1. CPAF mutant genotypes.
[A] Nucleotide polymorphisms revealed after whole genome sequence analysis for the isolated mutants. [B] Schematic of the early stop codons in the M532 and rst17 mutants.
